# Initiation of Antiseizure Medications in Patients With Brain Abscess

**DOI:** 10.1001/jamanetworkopen.2025.24557

**Published:** 2025-08-01

**Authors:** Victoria M. Nielsen, Michael Klompas, Justin Manjourides, Louisa H. Smith

**Affiliations:** 1 Department of Public Health and Health Sciences, Northeastern University, Boston, Massachusetts; 2 Department of Population Medicine, Harvard Medical School and Harvard Pilgrim Health Care Institute, Boston, Massachusetts; 3 Department of Medicine, Brigham and Women’s Hospital, Boston, Massachusetts; 4 Roux Institute at Northeastern University, Portland, Maine

## Abstract

**Importance:**

Epilepsy is a common complication of brain abscess. However, the effectiveness of antiseizure medications (ASMs) in preventing epilepsy in brain abscess survivors is unknown.

**Objective:**

To assess whether the initiation of ASMs is associated with a reduced risk of epilepsy.

**Design, Setting, and Participants:**

This retrospective cohort study conducted a target trial emulation using US commercial insurance claims data from October 1, 2016, to June 30, 2022, and followed up patients for 180 days. The study population was restricted to those with a diagnosis of brain abscess using *International Statistical Classification of Diseases, Tenth Revision, Clinical Modification* (*ICD-10-CM*) codes associated with an acute care visit. Only patients aged 18 years or older at the time of the brain abscess with at least 1 year of prior enrollment were included. Statistical analysis was performed from May to December 2024.

**Exposure:**

A priori selected study ASMs were levetiracetam, valproate, and phenytoin. A clone-censor-weight approach was used to compare the initiation of ASMs within a 45-day grace period (treatment arm) with no initiation of ASMs (control arm). Inverse probability weights were used to control for treatment selection.

**Main Outcomes and Measures:**

Study outcome was a diagnosis of epilepsy or seizure (with *ICD-10-CM* code) occurring 15 days or more after the index date. Weighted Kaplan-Meier models were fitted to estimate risk differences (RDs) at 90, 135, and 180 days accompanied by nonparametric bootstrapped 95% CIs. Sensitivity analyses were conducted to assess internal threats to validity.

**Results:**

Among the 572 patients included, the mean (SD) age was 61.5 (16.6) years and 353 (61.7%) were male. Of those in the treatment arm, 83 (88.3%) initiated ASMs within the first 30 days. Overall, 129 patients (22.5%) developed epilepsy during follow-up. There was no statistically significant risk difference in the probability of epilepsy incidence at each follow-up time point (RD at 90 days, –0.02% [95% CI, −4.9% to 4.8%]; RD at 135 days, 1.9% [95% CI, −5.0% to 8.5%]; RD at 180 days, 3.5% [95% CI, –4.4% to 10.8%]). Sensitivity analyses agreed with the primary findings.

**Conclusions and Relevance:**

In this cohort study of brain abscess survivors, initiation of ASMs was not associated with a reduced risk of epilepsy. Future studies should replicate the findings and consider alternative treatment protocols.

## Key Points

**Question:**

Are antiseizure medications (ASMs) associated with a reduced risk of epilepsy in brain abscess survivors at 90, 135, and 180 days of follow-up?

**Findings:**

In this cohort study of 572 patients with a brain abscess, there were no statistically significant differences in the probabilities of epilepsy occurrence at 90, 135, and 180 days after initiation of ASMs.

**Meaning:**

The findings suggest that the initiation of ASMs was not associated with reduced epilepsy risk in patients with brain abscess.

## Teaser

This cohort study assesses whether antiseizure medications are associated with epilepsy risk among patients with brain abcess.

## Introduction

Brain abscess consists of an encapsulated area of pus within the brain, often resulting from an infectious agent.^1^ There are approximately 1500 to 2500 brain abscesses diagnosed in the US annually.^1^ Albeit an infrequent occurrence, brain abscess is associated with numerous sequelae.^1,2,3,4^

Epilepsy—the occurrence of repeated unprovoked seizures—is a common complication of brain abscess.^5,6,7,8^ Epilepsy has social, medical, and economic implications, such as experiences of stigma and increased mortality risk.^9^ Taken with its common occurrence in brain abscess survivors, treatments that prevent development of seizures and epilepsy should be identified.^4,5,10,11^ However, literature on the topic is limited.^4^

The European Society of Clinical Microbiology and Infectious Diseases released clinical guidelines for the management of brain abscess, including whether antiseizure medications (ASMs) should be initiated to prevent epilepsy.^4^ The guidelines state that ASMs should not be initiated given the scant literature on the topic.^4^ To our knowledge, there are no randomized clinical trials (RCTs) assessing the effectiveness of ASMs in preventing epilepsy in survivors of brain abscess.^4^ Nevertheless, in clinical practice, ASMs may be prescribed preventively.^12^

We used a target trial emulation to assess whether initiation of ASMs is associated with a reduced risk of epilepsy in patients with brain abscess.^13,14^ This framework aligns analysis of observational data with a hypothetical RCT and rigorously accounts for biases that arise in the analysis of observational data.^13,14^ We hypothesized that ASMs are associated with a reduced risk of epilepsy in brain abscess survivors.

## Methods

In this retrospective cohort study, we designed an ideal RCT to answer the question of interest: is initiation of ASMs within 45 days of presentation with brain abscess associated with reduced incidence of epilepsy? (Table 1). We then emulated this target trial using US commercial claims data. The Northeastern University Institutional Review Board deemed this study exempt from review because it was not human participant research. We followed the Strengthening the Reporting of Observational Studies in Epidemiology (STROBE) reporting guideline.^15^

**Table 1.** Target Trial and Emulation Using Observational Data

Components	Target trial	Emulated trial
Aim	To answer the question of interest: is initiation of ASMs within 45 d of presentation with brain abscess associated with reduced incidence of epilepsy?	Same objective
Study design and data source	RCT	Observational equivalent of an RCT
Eligibility criteria	Aged ≥18 y at time of brain abscess; no history of epilepsy or seizures; no prior use of levetiracetam, valproate, and phenytoin; hospitalized with brain abscess	Same age; same disease history, excluding diagnoses with *ICD-10-CM* codes ≥30 d before index date; same medication history; same *ICD-10-CM* code for brain abscess with visit type of hospitalization, emergency department, or urgent care; 1-y continuous prior enrollment
Treatment strategies	Two study arms: initiation of ASMs within 45 d of baseline (treatment arm), and no initiation of ASMs (control arm)	Same arms
Treatment assignment	Randomization	Self-selection into treatment: initiation of ASMs within 45 d of the index date vs no initiation of ASMs within 45 d of index date
Follow-up	180 d	Same follow-up duration
Outcome	Epilepsy	Same outcome, including events occurring only ≥15 d after index date
Causal contrast	Risk differences at 90, 135, and 180 d after baseline	Same risk differences
Analysis plan	Kaplan-Meier analysis	Clone-censor-weight strategy, in which observations are assumed to follow both treatment strategies until they are observed to deviate from 1 strategy, at which point they are censored. An inverse probability–weighted Kaplan-Meier analysis adjusts for selection into treatment. See statistical analysis plan.

Abbreviations: ASM, antiseizure medication; *ICD-10-CM, International Statistical Classification of Diseases, Tenth Revision, Clinical Modification*; RCT, randomized clinical trial.

### Population

We used IQVIA PharMetrics Plus for Academics claims data normalized to the Observational Medical Outcomes Partnership Common Data Model.^16,17^ PharMetrics contains adjudicated commercial insurance claims on 62.7 million patients in the US, including detailed pharmacy, procedures, health care utilization, and medical history. Health care claims between October 1, 2016, and June 30, 2022, were included.

Our population was restricted to patients with a diagnosis of brain abscess identified using *International Statistical Classification of Diseases, Tenth Revision, Clinical Modification* (*ICD-10-CM*) code G06.0 (eTable 1 in Supplement 1). Given that a brain abscess is a medical emergency, we defined the index event as an abscess associated with an acute care visit type based on Centers for Medicare and Medicaid Services Place of Service code to exclude cases that represented management of a prior abscess (eTable 2 in Supplement 1). Only patients aged 18 years or older at the time of the abscess with at least 1 year of prior enrollment captured in PharMetrics were included. We excluded patients receiving the study ASMs prior to the index date (eTable 2 in Supplement 1).

Patients with preexisting diagnosis codes of epilepsy or convulsions 30 days or more prior to the index date were also excluded (eTable 1 in Supplement 1). We elected to use a 30-day window, as seizures occurring in close temporality to the brain abscess may be symptomatic of the abscess.^3,5,18^ We did not exclude patients with a prescription claim for non–study ASMs but no epilepsy diagnosis due to frequent off-label use of these drugs.

Date of brain abscess was assigned as the index date. We followed up patients for 180 days after the index date or until loss to follow-up.

### Treatment, Outcome, and Independent Variables

We defined 2 treatment strategies: (1) initiation of ASMs within a 45-day grace period after the index date (treatment arm) and (2) no initiation of ASMs (control arm). We a priori selected levetiracetam, valproate, and phenytoin as the study ASMs because these medications are often the first choice in preventing seizures in patients with brain abscess (eTable 2 in Supplement 1). Drug data in the Observational Medical Outcomes Partnership database, including brand names and manufacturers, are standardized and identifiable by a single concept identification.^19^ For ASM initiation, we elected to use a longer grace period as limited guidance on this topic may result in heterogeneity in timing of ASM initiation.

Whenever possible, we used previously defined metrics in claims or other clinical datasets, including our outcome definition.^5,7,10,20,21,22,23,24,25,26^ We also followed best-practice guidelines of analysis of claims data, including limiting analysis to broad groups of service codes and limiting covariables to those better captured in claims data.^27^

We defined our study outcome as a diagnosis of epilepsy or seizures occurring at least 15 days after the index date to ensure that early provoked seizures were not classified as epilepsy.^12^ We also considered a 30-day interval as a sensitivity analysis.^5,10^ To determine which variables needed to be adjusted for in our analysis, we created a directed acyclic graph to clarify the causal association of our study question (eFigure 1 in Supplement 1). Confounders biasing the pathway between ASMs and the outcome were included as covariables in the model.

We defined clinical concepts based on *ICD-10-CM*, RxNorm, and *Current Procedural Terminology* codes. Each covariable was classified as baseline (non–time varying) or time varying. Baseline variables included medical history of traumatic brain injury, congenital heart condition, neurosurgery, stroke, alcohol misuse, and brain cancer.^2,4,5,10,20^ We included age (years), sex (male and female), US region (North, South, Midwest, and West), Charlson Comorbidity Index (range: 0-24, with the highest index indicating the highest number of comorbidities), sepsis, and critical illness.^22,23,24^ Time-varying confounders included neurosurgical management of abscess (craniotomy and aspiration, defined as 2 separate covariables). We controlled for early seizures, defined as a seizure occurring within 29 days prior to and within 14 days after the index date.^10,12,18^

### Statistical Analysis

In a clinical trial, randomization would achieve balance of confounders. In our emulation, we balanced measured confounders by cloning patients into both treatment and control arms to achieve baseline balance and by using inverse probability weights to re-weight the study arms during the 45-day grace period to adjust for artificial censoring due to nonadherence to a given treatment strategy.^14,28^ This approach results in all patients contributing data to both arms if they did not survive without the outcome long enough to begin treatment, thus addressing immortal time bias (for example, a patient who had a seizure on day 31 but started the drug on day 33 would contribute to both study arms).^14,28^ Once the 45-day grace period is over and if patients remain uncensored in their respective study arm, they contribute follow-up to the subsequent outcome model.

We identified when a clone patient’s observed treatment strategy no longer aligned with their assigned treatment arm and censored the observation at that point. This strategy was defined in the control arm as the initiation of an ASM within the 45-day grace period (date of censoring set as the date of ASM initiation) and in the treatment arm as the completion of the 45-day grace period without starting an ASM (date of censoring set at 45 days). We then fit a logistic regression model for the treatment group and a Cox proportional hazards regression model for the control group using censoring as the event to estimate the probability of censoring during the 45-day grace period, separately within the treatment and control arms, and we used these models to generate inverse probability weights. We assessed model weights for extreme weights and compared the weight distribution between each arm to ensure adequate overlap and to evaluate positivity violations. We then applied the weights and estimated standardized mean difference (SMD) between treatment groups for all covariables to assess balance.

We then fit weighted Kaplan-Meier models to obtain probabilities of developing epilepsy at 90 days, 135 days, and 180 days for the treatment and control arms. We subtracted the treatment arm probability from the control arm probability to calculate the marginal risk difference (RD) at 90 days, 135 days, and 180 days. We assessed statistical significance using 95% confidence intervals of the risk differences. To obtain 95% CIs, we performed nonparametric bootstrapping with 1000 replicates.

To assess internal threats to validity, we conducted 3 sensitivity analyses. First, we redefined seizure occurrence as those occurring only 30 days or more after the index date to determine whether potential misclassification of early provoked seizures biased our study findings.^5,10^ For this analysis, early seizures were redefined as those occurring within 29 days prior to 29 days after the index date. Second, we lengthened the grace period to 90 days from 45 days to allow for later initiation of ASMs in the treatment arm. Third, we conducted a negative control study (in which no association between exposure and outcome was expected) using infectious pneumonia occurring 15 days or more after the index date to detect unmeasured confounding. We would expect no differences between groups because ASMs are not associated with infectious pneumonia.

The eMethods and eFigure 2 in Supplement 1 provide a full description of the analytic plan. Data analysis was performed from May to December 2024 using RStudio, version 4.3.1 (R Project for Statistical Computing).

## Results

Of the 1623 patients with cerebral abscesses during the study period, 572 were eligible for our analysis. These patients had a mean (SD) age of 61.5 (16.6) years and included 219 females (38.3%) and 353 males (61.7%) (Table 2). Two hundred twenty-four patients (39.2%) had their abscess managed with aspiration, and 196 (34.2%) had their abscess managed with craniotomy. The most common medical history recorded included neurosurgery (215 [37.6%]) and stroke (159 [27.8%]). Most patients were living in the Southern region (163 [28.5%]).

**Table 2.** Descriptive Statistics of Patients With Brain Abscess (N = 572)

Characteristic	Patients, No. (%)		SMD^a^	
	No initiation of ASMs (n = 478)	Initiation of ASMs (n = 94)	Preweighting	Postweighting
Sex				
Female	192 (40.2)	27 (28.7)	0.243	0.006
Male	286 (59.8)	67 (71.3)	1 [Reference]	1 [Reference]
Age, mean (SD), y	63.3 (15.5)	52.8 (18.9)	0.606	0.012
Clinical course				
Critical illness	206 (43.1)	50 (53.2)	0.203	<0.001
Sepsis	154 (32.2)	21 (22.3)	0.223	0.010
Aspiration of abscess	170 (35.6)	54 (57.4)	0.450	0.008
Craniotomy of abscess	152 (31.8)	44 (46.8)	0.311	0.019
Early seizure	47 (9.8)	18 (19.1)	0.267	0.010
Medical history				
Neurosurgery	192 (40.2)	23 (24.5)	0.341	0.007
Stroke	141 (29.5)	18 (19.1)	0.243	<0.001
TBI	34 (7.1)	7 (7.4)	0.013	0.004
Alcohol misuse	30 (6.3)	6 (6.4)	0.004	0.014
Brain cancer	61 (12.8)	9 (9.6)	0.101	0.008
CHD	11 (2.3)	<5^b^	0.012	0.004
US region				
Midwest	124 (25.9)	31 (33.0)	0.155	<0.001
North	85 (17.8)	17 (18.1)	0.008	0.007
South	136 (28.5)	27 (28.7)	0.006	0.004
West	133 (27.8)	19 (20.2)	0.179	0.010
CCI, mean (SD)	2.8 (3.2)	1.8 (2.6)	0.346	0.006

Abbreviations: ASM, antiseizure medication; CCI, Charlson Comorbidity Index; CHD, congenital heart disease; SMD, standardized mean difference; TBI, traumatic brain injury.

^a^ SMD at baseline, calculated as the square root of the Mahalanobis distance.

^b^ Suppressed to protect patient confidentiality.

Patients were excluded due to the following reasons (not mutually exclusive): 512 had an index event not associated with an acute care visit, 116 were younger than 18 years, 185 had a past diagnosis of epilepsy, and 172 had prior use of study ASMs. Only 8 patients were excluded due to missing data (missing geography). Because the study period overlapped with the COVID-19 pandemic, we assessed temporal trends in brain abscess, epilepsy, and ASM initiation in our study population. We did not find any notable shifts before or after the pandemic.

In our study population, 129 (22.5%) experienced a seizure 15 to 180 days after the index date. The median (IQR) days to seizure from the index date were 32 (18-54) days. Ninety-four patients (16.4%) initiated ASMs during the 45-day grace period, and 478 (83.6%) did not initiate ASMs. The most common drug prescribed was levetiracetam (n = 89), followed by phenytoin (n = 8) and valproate (n = <5). The median (IQR) daily doses for each drug were 1500 (1000-2000) mg for levetiracetam, 300 (90-500) mg for phenytoin, and 1000 (500-1200) mg for valproate. Of these patients, 7 (7.4%) were prescribed a combination of phenytoin and levetiracetam or a combination of valproate and levetiracetam. No patients were prescribed all 3 medications. The median (IQR) days to initiation of the drug from the index date were 9 (4.0-17.7) days. The majority of patients initiated the ASM within the first 30 days (83 [88.3%]). The median (IQR) duration of the ASM was 29 (14-29) days. Among those who initiated an ASM, 71 (75.5%) were prescribed the drug for 14 days or more.

Prior to weighting, there were notable differences between patients initiating (treatment arm) vs not initiating ASMs (control arm) (Table 2). Those in the treatment group compared with the control group were less often female (27 [28.7%] vs 192 [40.2%]), younger (mean [SD] age, 52.8 [18.9] years vs 63.3 [15.5] years), more often had their abscess neurosurgically managed (aspiration: 54 [57.4%] vs 170 [35.6%]; craniotomy: 44 [46.8%] vs 152 [31.8%]), more often had early seizures (18 [19.1%] vs 47 [9.8%]), and had a lower Charlson Comorbidity Index (1.8 [2.6] vs 2.8 [3.2]). Before weighting, the maximum SMD was 0.606 (age).

The logistic regression for the treatment group indicated no model violations. The Cox proportional hazards regression model for the control group indicated violations of proportional hazards for critical illness, stroke, and craniotomy of the abscess. We thus stratified based on these covariables and also included an interaction term between the critical illness stratum and aspiration of abscess based on Akaike information criterion. Inverse probability weights were similar between the 2 study arms, with good overlap and no extreme weights. After weighting, differences between groups were markedly reduced (Table 2).

The probabilities of epilepsy occurrence 15 days or more after the index date in the treatment and control arms during follow-up were not statistically significantly different (a positive value indicating a higher probability in the treatment arm). Both groups experienced similar probabilities of epilepsy occurrence (RD at 90-day follow-up, –0.02% [95% CI, −4.9% to 4.8%]; RD at 135-day follow-up, 1.9% [95% CI, −5.0% to 8.5%]; RD at 180-day follow-up, 3.5% [95% CI, –4.4% to 10.8%]). Table 3 shows study results and the Figure provides survival curves of the treatment and control arms. Among 32 patients who initiated an ASM, 13 (40.6%) were still filling an ASM prescription when the outcome occurred. These patients had been using the medication for a median (IQR) of 14 (9-19) days when the outcome event occurred.

**Table 3.** Probability of Epilepsy Occurrence During Follow-Up Among Treatment and Control Arms for Main Outcome and Sensitivity Analyses (N = 572)

Model	Epilepsy occurrence % (95% CI)			
	15-180 d	30-180 d	90-d Grace period	Negative control^a^
**90 d of follow-up**				
Treatment arm	20.7 (15.7 to 25.7)	8.6 (4.3 to 12.4)	21.8 (18.1 to 25.4)	12.1 (7.2 to 17.2)
Control arm	20.8 (16.8 to 24.7)	10.7 (7.4 to 14.0)	20.5 (16.5 to 24.3)	14.6 (11.1 to 18.2)
RD	−0.02 (−4.9 to 4.8)	−2.0 (−6.8 to 2.1)	1.3 (−0.6 to 3.3)	−2.4 (−7.3 to 2.4)
**135 d of follow-up**				
Treatment arm	25.4 (18.6 to 31.7)	12.6 (6.6 to 18.1)	26.3 (20.2 to 31.7)	14.1 (8.4 to 19.9)
Control arm	23.4 (19.1 to 27.6)	13.4 (9.8 to 17.0)	23.3 (19.0 to 27.3)	16.4 (12.7 to 20.2)
RD	1.9 (−5.0 to 8.5)	−0.7 (−7.3 to 5.2)	3.0 (−2.6 to 8.1)	−2.2 (−8.1 to 3.4)
**180 d of follow-up**				
Treatment arm	27.7 (20.0 to 34.7)	14.9 (7.9 to 21.3)	28.4 (21.4 to 34.6)	18.4 (10.2 to 26.8)
Control arm	24.2 (19.8 to 28.5)	14.1 (10.5 to 17.9)	24.1 (19.7 to 28.3)	19.7 (15.7 to 23.7)
RD	3.5 (−4.4 to 10.8)	0.7 (−6.8 to 7.7)	4.3 (−2.3 to 10.4)	−1.2 (−9.7 to 7.4)

Abbreviation: RD, risk difference.

^a^ Outcome for the negative control study was infectious pneumonia.

**Figure.**
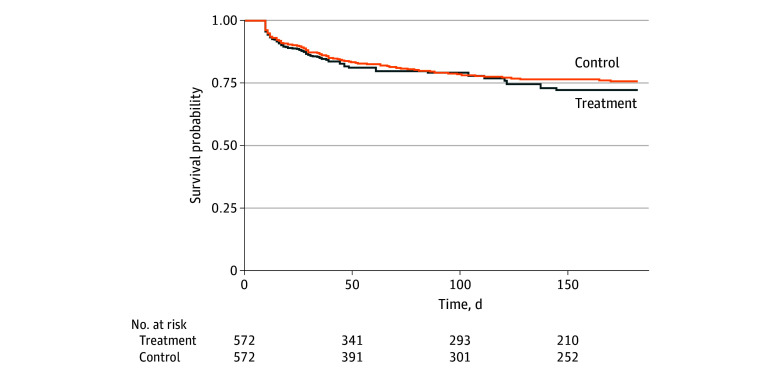
Survival Curves of Seizures by Treatment and Control Arms With a 45-Day Grace Period Inverse probability weighted survival curves were derived from the Kaplan-Meier model of cloned patients with brain abscess in the treatment and control arms.

Sensitivity analyses agreed with our primary finding (Table 3). Using a 30-day window to assess potential misclassification of early seizures, we found no differences between study arms (RD at 90-day follow-up, −2.0% [95% CI, −6.8% to 2.1%]; RD at 135-day follow-up, −0.7% [95% CI, –7.3% to 5.2%]; RD at 180-day follow-up, 0.7% [95% CI, −6.8% to 7.7%]). Increasing the grace period to 90 days also agreed with our primary study findings (RD at 90-day follow-up, 1.3% [95% CI, −0.6% to 3.3%]; RD at 135-day follow-up, 3.0% [95% CI, –2.6% to 8.1%]; RD at 180-day follow-up, 4.3% [95% CI, −2.3% to 10.4%]). Finally, there were no differences between groups in the negative control study (RD at 90-day follow-up, −2.4% [95% CI, −7.3% to 2.4%]; RD at 135-day follow-up, −2.2% [95% CI, –8.1% to 3.4%]; RD at 180-day follow-up, −1.2% [95% CI, −9.7% to 7.4%]).

## Discussion

We did not find ASMs to be associated with a reduced incidence of epilepsy among brain abscess survivors. Our findings were robust against internal threats to validity, as evidenced through multiple sensitivity analyses corroborating the primary study finding. We believe that our findings have important implications for future research on ASMs.

The 180-day incidence of epilepsy in our study population (22.5%) was similar to a recent study that reported 27.3% of survivors developed epilepsy approximately 9 months after the abscess.^10^ Furthermore, our results agree with findings of similar studies on conditions theorized to cause epilepsy. A meta-analysis of initiation of ASMs as prophylaxis of early seizure in patients with traumatic brain injury, which included 3 RCTs, found only modest evidence of a protective effect against seizures.^29^ Another meta-analysis of 2 RCTs and 4 observational studies of patients with de novo brain tumor after craniotomy did not find benefit of administration of ASMs in preventing epilepsy.^30^ Another review of clinical trials of ASM prophylaxis in brain tumors did not find evidence for or against initiation of ASMs in this population.^31^ A study of initiation of seizure prophylaxis in survivors of acute ischemic stroke did not find a benefit in reducing 30-day mortality.^32^ Finally, a study of initiation of corticosteroids in patients with neurosurgically treated brain abscess did not find a protective effect in late unprovoked seizures.^12^ This same study paradoxically found an increased incidence of late unprovoked seizures in patients who received ASMs, likely due to confounding.^4,12^

Brain abscess is an infrequent occurrence, and leveraging clinical data with large coverage can ensure a large enough sample size for sufficient statistical power and increase generalizability.^33,34^ However, our analysis does not definitively settle the question of the role of ASMs in preventing epilepsy. Due to the scant literature on the topic, we designed the question of interest broadly. Future studies can include per protocol analyses of more specific treatment regimens, such as different drug doses, alternative ASMs, and various combinations of ASMs. Furthermore, numerous studies have assessed whether different types of neurosurgical management, causative microbial agents, or size and location of the abscess affect subsequent development of epilepsy.^10,12,35^ Heterogeneity in the risk of subsequent epilepsy and seizures based on the clinical presentation may have implications for treatment effectiveness.^10,12,35^

In this same vein, the duration of ASM therapy was 3 to 4 weeks in our population. Patients may require a longer regimen prior to discontinuation of the ASM to prevent the development of epilepsy. Although 1 study suggests that most cases of epilepsy develop within the first year after the brain abscess, cases can occur up to 10 years later.^10^ However, in our study, nearly half of the population in the treatment arm who had the outcome had a filled ASM prescription and had been using the drug for 14 days when the outcome occurred. Given this finding, a longer duration of therapy must be weighed against the costs and burden of unnecessary treatment.^4^

### Limitations

Claims data are driven by billing practices and may not fully represent the clinical course of the patient. However, using previously defined metrics and analyzing broad categories of service codes in alignment with best practices of claims data analysis strengthen internal validity. Furthermore, we argue that the study outcome (epilepsy) will be well-captured in our study population due to clinical recommendations that brain abscess survivors and their caregivers be educated on symptoms of increased intracranial pressure (including seizures) and the necessity of seeking medical care if such symptoms occur.^1^ PharMetrics contains only commercial claims data, which may represent a healthier and younger subset of brain abscess cases with better health care access. Therefore, our population may have different outcomes than patients with lower income and patients aged 65 years or older. Although the inclusion of covariables in the model was comprehensive and based on a thorough review of the literature, the absence of residual confounding cannot be empirically verified. Finally, given that this work is an observational study, our analysis was limited to current clinical practice. A longer duration of ASM therapy may be necessary to prevent epilepsy occurrence in brain abscess survivors.

## Conclusions

In this cohort study of brain abscess survivors, the emulated trial did not find the initiation of ASMs to be associated with a reduction in epilepsy risk. Future RCTs and observational studies should attempt to replicate the study findings and consider alternative treatment regimens, especially those focused on longer durations of ASM therapy.

## Notes

Supplement 1. **eTable 1.** Concept Definitions Using Source Codes **eTable 2.** OMOP Concepts **eFigure 1.** Directed Acyclic Graph **eMethods.** Detailed Statistical Analysis Plan **eFigure 2.** Inverse Probability Weights in Treatment and Control Arms

Supplement 2.
Data Sharing Statement

## References

[1] Hall WA, Mesfin FB. *Brain Abscess*. StatPearls Publishing; 2024. Accessed July 19, 2024. https://www.ncbi.nlm.nih.gov/books/NBK441841/28722871

[2] Brouwer MC, Coutinho JM, van de Beek D. Clinical characteristics and outcome of brain abscess: systematic review and meta-analysis. Neurology. 2014;82(9):806-813. doi:10.1212/WNL.0000000000000172 24477107

[3] Brouwer MC, Tunkel AR, McKhann GM II, van de Beek D. Brain abscess. N Engl J Med. 2014;371(5):447-456. doi:10.1056/NEJMra1301635 25075836

[4] Bodilsen J, D’Alessandris QG, Humphreys H, ; ESCMID Study Group for Infections of the Brain (ESGIB). European Society of Clinical Microbiology and Infectious Diseases guidelines on diagnosis and treatment of brain abscess in children and adults. Clin Microbiol Infect. 2024;30(1):66-89. doi:10.1016/j.cmi.2023.08.016 37648062

[5] Bodilsen J, Dalager-Pedersen M, van de Beek D, Brouwer MC, Nielsen H. Long-term mortality and epilepsy in patients after brain abscess: a nationwide population-based matched cohort study. Clin Infect Dis. 2020;71(11):2825-2832. doi:10.1093/cid/ciz1153 31773138

[6] Kilpatrick C. Epilepsy and brain abscess. J Clin Neurosci. 1997;4(1):26-28. doi:10.1016/S0967-5868(97)90006-0 18638919

[7] Zelano J, Westman G. Epilepsy after brain infection in adults: a register-based population-wide study. Neurology. 2020;95(24):e3213-e3220. doi:10.1212/WNL.0000000000010954 32989110

[8] Vezzani A, Fujinami RS, White HS, . Infections, inflammation and epilepsy. Acta Neuropathol. 2016;131(2):211-234. doi:10.1007/s00401-015-1481-5 26423537 PMC4867498

[9] Falip M, Gil-Nagel A, Viteri Torres C, Gómez-Alonso J. Diagnostic problems in the initial assessment of epilepsy. Neurologist. 2007;13(6, suppl 1):S2-S10. doi:10.1097/NRL.0b013e31815bb11b 18090947

[10] Bodilsen J, Duerlund LS, Mariager T, ; for DASGIB. Risk factors and prognosis of epilepsy following brain abscess: a nationwide population-based cohort study. Neurology. 2023;100(15):e1611-e1620. doi:10.1212/WNL.0000000000206866 36810235 PMC10103119

[11] Ito H. Epilepsy prevention after brain abscess: is it time to rethink the indication? Clin Infect Dis. 2021;73(5):939. doi:10.1093/cid/ciab163 33623998

[12] Lee HS, Kim JH, Kim YH, Lee S. Predictors of unprovoked seizures in surgically treated pyogenic brain abscess: does perioperative adjunctive use of steroids has any protective effect? Clin Neurol Neurosurg. 2018;173:46-51. doi:10.1016/j.clineuro.2018.07.024 30086427

[13] Hernán MA, Robins JM. Using big data to emulate a target trial when a randomized trial is not available. Am J Epidemiol. 2016;183(8):758-764. doi:10.1093/aje/kwv254 26994063 PMC4832051

[14] Hernán MA, Sauer BC, Hernández-Díaz S, Platt R, Shrier I. Specifying a target trial prevents immortal time bias and other self-inflicted injuries in observational analyses. J Clin Epidemiol. 2016;79:70-75. doi:10.1016/j.jclinepi.2016.04.014 27237061 PMC5124536

[15] von Elm E, Altman DG, Egger M, Pocock SJ, Gotzsche PC, Vandenbroucke JP. The Strengthening the Reporting of Observational Studies in Epidemiology (STROBE) Statement: guidelines for reporting observational studies. Accessed January 1, 2025. https://www.equator-network.org/reporting-guidelines/strobe/

[16] Observational Health Data Sciences and Informatics. Accessed July 19, 2024. https://www.ohdsi.org/

[17] IQVIA PharMetrics Plus. Accessed July 19, 2024. https://www.iqvia.com/locations/united-states/library/fact-sheets/iqvia-pharmetrics-plus

[18] Chuang MJ, Chang WN, Chang HW, . Predictors and long-term outcome of seizures after bacterial brain abscess. J Neurol Neurosurg Psychiatry. 2010;81(8):913-917. doi:10.1136/jnnp.2009.195073 20682720

[19] Athena. Accessed July 19, 2024. https://athena.ohdsi.org/search-terms/start

[20] Bodilsen J, Dalager-Pedersen M, van de Beek D, Brouwer MC, Nielsen H. Risk factors for brain abscess: a nationwide, population-based, nested case-control study. Clin Infect Dis. 2020;71(4):1040-1046. doi:10.1093/cid/ciz890 31641757

[21] Bodilsen J, Søgaard KK, Nielsen H, Omland LH. Brain abscess and risk of cancer: a nationwide population-based cohort study. Neurology. 2022;99(8):e835-e842. doi:10.1212/WNL.0000000000200769 35995592

[22] Charlson ME, Pompei P, Ales KL, MacKenzie CR. A new method of classifying prognostic comorbidity in longitudinal studies: development and validation. J Chronic Dis. 1987;40(5):373-383. doi:10.1016/0021-9681(87)90171-8 3558716

[23] Quan H, Sundararajan V, Halfon P, . Coding algorithms for defining comorbidities in ICD-9-CM and ICD-10 administrative data. Med Care. 2005;43(11):1130-1139. doi:10.1097/01.mlr.0000182534.19832.83 16224307

[24] Quan H, Li B, Couris CM, . Updating and validating the Charlson Comorbidity Index and score for risk adjustment in hospital discharge abstracts using data from 6 countries. Am J Epidemiol. 2011;173(6):676-682. doi:10.1093/aje/kwq433 21330339

[25] Jetté N, Reid AY, Quan H, Hill MD, Wiebe S. How accurate is ICD coding for epilepsy? Epilepsia. 2010;51(1):62-69. doi:10.1111/j.1528-1167.2009.02201.x 19682027

[26] Peterson A, Gabella BA, Johnson J, . Multisite medical record review of emergency department visits for unspecified injury of head following the ICD-10-CM coding transition. Inj Prev. 2021;27(S1):i13-i18. doi:10.1136/injuryprev-2019-043517 33674328 PMC7948189

[27] Konrad R, Zhang W, Bjarndóttir M, Proaño R. Key considerations when using health insurance claims data in advanced data analyses: an experience report. Health Syst (Basingstoke). 2019;9(4):317-325. doi:10.1080/20476965.2019.1581433 33354323 PMC7738306

[28] Maringe C, Benitez Majano S, Exarchakou A, . Reflection on modern methods: trial emulation in the presence of immortal-time bias—assessing the benefit of major surgery for elderly lung cancer patients using observational data. Int J Epidemiol. 2020;49(5):1719-1729. doi:10.1093/ije/dyaa057 32386426 PMC7823243

[29] Wat R, Mammi M, Paredes J, . The effectiveness of antiepileptic medications as prophylaxis of early seizure in patients with traumatic brain injury compared with placebo or no treatment: a systematic review and meta-analysis. World Neurosurg. 2019;122:433-440. doi:10.1016/j.wneu.2018.11.076 30465951

[30] Mirian C, Møller Pedersen M, Sabers A, Mathiesen T. Antiepileptic drugs as prophylaxis for de novo brain tumour-related epilepsy after craniotomy: a systematic review and meta-analysis of harm and benefits. J Neurol Neurosurg Psychiatry. 2019;90(5):599-607. doi:10.1136/jnnp-2018-319609 30674543

[31] Tremont-Lukats IW, Ratilal BO, Armstrong T, Gilbert MR. Antiepileptic drugs for preventing seizures in people with brain tumors. Cochrane Database Syst Rev. 2008;2008(2):CD004424. doi:10.1002/14651858.CD004424.pub2 18425902 PMC9036944

[32] Moura LMVR, Donahue MA, Yan Z, . Comparative effectiveness and safety of seizure prophylaxis among adults after acute ischemic stroke. Stroke. 2023;54(2):527-536. doi:10.1161/STROKEAHA.122.039946 36544249 PMC9870933

[33] Reich C, Ostropolets A, Ryan P, . OHDSI standardized vocabularies-a large-scale centralized reference ontology for international data harmonization. J Am Med Inform Assoc. 2024;31(3):583-590. doi:10.1093/jamia/ocad247 38175665 PMC10873827

[34] Coloma PM, Trifirò G, Schuemie MJ, ; EU-ADR Consortium. Electronic healthcare databases for active drug safety surveillance: is there enough leverage? Pharmacoepidemiol Drug Saf. 2012;21(6):611-621. doi:10.1002/pds.3197 22315152

[35] Helweg-Larsen J, Astradsson A, Richhall H, Erdal J, Laursen A, Brennum J. Pyogenic brain abscess: a 15 year survey. BMC Infect Dis. 2012;12:332. doi:10.1186/1471-2334-12-332 23193986 PMC3536615

