# Supplementary material for: Initiation of Antiseizure Medications in Patients With Brain Abscess
Source: JAMA Netw Open. 2025 Aug 1;8(8):e2524557. doi: 10.1001/jamanetworkopen.2025.24557 (PMC12317356; doi:10.1001/jamanetworkopen.2025.24557)
Supplement: Supplement 1. — eTable 1. Concept Definitions Using Source Codes eTable 2. OMOP Concepts eFigure 1. Directed Acyclic Graph eMethods. Detailed Statistical Analysis Plan eFigure 2. Inverse Probability Weights in Treatment and Control Arms [file jamanetwopen-e2524557-s001.pdf]

Supplemental Online Content

Nielsen VM, Klompas M, Manjourides J, Smith LH. Initiation of antiseizure medications in patients with brain abscess. *JAMA Netw Open*. 2025;8(8):e2524557. doi:10.1001/jamanetworkopen.2025.24557

eTable 1. Concept Definitions Using Source Codes

eTable 2. OMOP Concepts

eFigure 1. Directed Acyclic Graph

eMethods. Detailed Statistical Analysis Plan

eFigure 2. Inverse Probability Weights in Treatment and Control Arms

This supplemental material has been provided by the authors to give readers additional information about their work.

© 2025 Nielsen VM et al. *JAMA Network Open*

eTable 1. Concept Definitions Using Source Codes.

Concept	Description	Codes	Vocabulary	Timeframe
Traumatic brain injury		S02.0, S02.1, S02.8, S02.91, S04.02, S04.03, S04.04, S06, S07.1, S09.90	ICD-10 CM	Any time prior to index date.
Congenital heart disease		Q20.0-Q20.8, Q21.0-Q21.8, Q22-Q24.8, Q25.0-Q25.8, Q26.0-Q26.4, Q26.8	ICD-10 CM	Any time prior to index date.
Stroke	Hemorrhagic, ischemic, or transient ischemic attack.	I60-I63, I65-I69, G45.9	ICD-10 CM	Any time prior to index date.
Early seizure	Seizures that are likely symptomatic of the brain abscess.	R56.8, G40	ICD-10 CM	Within 30 days prior to index date and within 14 days after index date.
Brain abscess	Index event.	G06.0	ICD-10 CM	Earliest 10/01/2016 to allow one-year look back prior to ICD-9 to 10 switch.
Epilepsy	Study outcome.	R56.8, G40	ICD-10 CM	Beginning 15 days after index date.
Alcohol misuse		F10, Z71.4, I42.6, K29.2, K70, G72.1, G62.1, K85.2, K86.0, E24.4, G31.2, Z72.1	ICD-10 CM	Any time prior to index date.
Brain cancer		C71; D33.0; D33.1; D33.2; D33.3; C79.3	ICD-10 CM	Any time prior to index date.
Sepsis		A02.1, A22.7, A26.7, A32.7, A40, A41.01, A41.02, A41.1, A41.2, A41.3, A41.4, A41.50, A41.51, A41.52, A41.53, A41.54, A41.59, A41.81, A41.89, A41.9, A42.7, A54.86, B37.7, O03.37, O03.87, O04.87, O07.37, O08.82, O85, O86.04, R65.2, T81.44	ICD-10 CM	Within 30 days before and 21 days after index date.

© 2025 Nielsen VM et al. *JAMA Network Open*

Concept	Description	Codes	Vocabulary	Timeframe
Critical illness	Includes treatment of vital organ failure or prevention of further life–threatening conditions. Delivering medical care in a moment of crisis, presence of a patient in an intensive care unit, or use of ventilation is not sufficient to bill a critical care (CC) service. Usually, a critical care service is provided to a patient in a "critical care area" such as the coronary care unit (CCU), ICU, respiratory care unit, or emergency room.	99291, 99292	CPT4	Within 30 days before and 21 days after index date.
Prior neurosurgery (excludes stereotactic radiosurgery)	Injection, Drainage, or Aspiration Procedures on the Skull, Meninges, and Brain.	61000-61070	CPT4	Not during same visit as brain abscess and at least 30 days prior to index date.
	Twist Drill, Burr Hole(s), or Trephine Procedures on the Skull, Meninges, and Brain.	61105-61253	CPT4	
	Craniectomy or Craniotomy Procedures.	61304-61576	CPT4	
	Anterior Cranial Fossa Skull Base Procedures.	61580-61586	CPT4	
	Middle Cranial Fossa Skull Base Procedures.	61590-61592	CPT4	
	Posterior Cranial Fossa Skull Base Procedures.	61595-61598	CPT4	
	Base of Anterior Cranial Fossa Procedures.	61600-61601	CPT4	
	Base of Middle Cranial Fossa Procedures.	61605-61613	CPT4	
	Base of Posterior Cranial Fossa Procedures.	61615-61616	CPT4	
	Repair and/or Reconstruction of Surgical Defects of Skull Base Procedures.	61618-61619	CPT4	
	Stereotaxis Procedures on the Skull, Meninges, and Brain.	61720-61791	CPT4	
	Endovascular Therapy Procedures on the Skull, Meninges, and Brain.	61623-61651	CPT4	
	Surgery for Aneurysm, Arteriovenous Malformation or Vascular Disease Procedures on the Skull, Meninges, and Brain.	61680-61711	CPT4	
	Neurostimulators (Intracranial) Procedures on the Skull,	61850-61892	CPT4	

Concept	Description	Codes	Vocabulary	Timeframe
	Meninges, and Brain.			
	Repair Procedures on the Skull, Meninges, and Brain.	62000-62148	CPT4	
	Neuroendoscopy Procedures on the Skull, Meninges, and Brain.	62160-62165	CPT4	
	Cerebrospinal Fluid (CSF) Shunt Procedures.	62180-62258	CPT4	
	Medical and Surgical, Central Nervous System and Cranial Nerves.	00*	ICD-10 PCS	
Charlson Comorbidity Index	Congestive Heart Failure (weight = 2).	I09.9, I11.0, I13.0, I13.2, I25.5, I42.0, I42.5-9, I43, I50, P29.0	ICD-10 CM	Any time prior to index date.
	Dementia (weight = 2).	F01-F03, F05.1, G30, G31.1	ICD-10 CM	
	Chronic Pulmonary Disease (weight = 1).	I27.8, 127.9, J40-J47, J60-J67, J68.4, J70.1, J70.3	ICD-10 CM	
	Rheumatic Disease (weight = 1).	M05, M06, M31.5, M32-M34, M35.1, M35.3, M36.0	ICD-10 CM	
	Mild Liver Disease (weight = 2).	B18, K70.0-K70.3, K70.9, K71.3-K71.5, K71.7, K73, K74, K76.0, K76.2-K76.4, K76.8, K76.9, Z94.4	ICD-10 CM	
	Diabetes W Complication (weight = 1).	E10.2-E10.5, E10.7, E11.2-E11.5, E11.7, E12.2-E12.5, E12.7, E13.2-E13.5, E13.7, E14.2-E14.5, E14.7	ICD-10 CM	
	Hemiplegia Or Paraplegia (weight = 2).	G04.1, G11.4, G80.1, G80.2, G81, G82, G83.0-G83.4, G83.9	ICD-10 CM	
	Renal Disease (weight = 1) See **Supplement 3** for codes used to identify dialysis.	I12.0, I13.1, N03.2-N03.7, N05.2-N05.7, N18, N19, N25.0, Z49.0-Z49.2, Z94.0, Z99.2	ICD-10 CM	
	Any Malignancy (weight = 2).	C00-C26, C30-C34, C37-C41, C43, C45-C58, C60-C76, C81-C85, C88, C90-C97	ICD-10 CM	
	Moderate Or Severe Liver Disease (weight = 4).	I85.0, I85.9, I86.4, 198.2, K70.4, K71.1, K72.1, K72.9, K76.5, K76.6, K76.7	ICD-10 CM	
	Metastatic Solid Tumor (weight = 6).	C77-C80	ICD-10 CM	

© 2025 Nielsen VM et al. *JAMA Network Open*

Concept	Description	Codes	Vocabulary	Timeframe
	AIDS/HIV (weight = 4).	B20-B22, B24	ICD-10 CM	
Neurosurgical management: Aspiration	Injection, Drainage, or Aspiration Procedures on the Skull, Meninges, and Brain.	61000-61070	CPT4	On or after date of brain abscess.
	Twist Drill, Burr Hole(s), or Trephine Procedures on the Skull, Meninges, and Brain.	61105-61253	CPT4	
	Stereotaxis Procedures on the Skull, Meninges, and Brain.	61720-61791	CPT4	
	Medical and Surgical, Central Nervous System and Cranial Nerves, Drainage.	009*	ICD-10 PCS	
Neurosurgical management: Craniotomy or Craniectomy	Craniectomy or Craniotomy Procedures.	61304-61576	CPT4	On or after date of brain abscess.
	Medical and Surgical, Central Nervous System and Cranial Nerves, Excision.	00B*	ICD-10 PCS	
	Medical and Surgical, Head and Facial Bones, Excision.	0NB*	ICD-10 PCS	
Infectious pneumonia	Alternative outcome for sensitivity analysis.	A01.03, A02.22, A37.01, A37.11, A37.81, A37.91, A40.3, A54.84, B01.2, B05.2, B06.81, B77.81, B95.3, B96.0, B96.1, J09.X1, J10.00, J10.01, J10.08, J11.00, J11.08, J12.0, J12.1, J12.2, J12.3, J12.81, J12.82, J12.89, J12.9, J13, J14, J15.0, J15.1, J15.20, J15.211, J15.212, J15.29, J15.3, J15.4, J15.5, J15.61, J15.69, J15.7, J15.8, J15.9, J16.0, J16.8, J17, J18.0, J18.1, J18.2, J18.8, J18.9, J20.0, J85.1, J95.851	ICD-10 CM	Beginning 15 days after index date.

© 2025 Nielsen VM et al. *JAMA Network Open*

eTable 2. OMOP Concepts.

Concept	Description	Concept IDs	Domain ID	Vocabulary	Timeframe
Acute care encounter.	Inpatient hospital, urgent care facility, emergency room, or observation room.	8717, 8782, 8870, 9201, 581385	Visit	CMS Place of Service	Visit type associated with brain abscess diagnosis code.
Dialysis services and procedures.	Receipt of dialysis (for calculation of Charlson Comorbidity Index).	45889365	Observation	CPT4	Any time prior to brain abscess.
Levetiracetam, Valproate, and Phenytoin.	Study drugs.	711584, 74546, 740910	Drug	RxNorm	Within 45-days of brain abscess.

© 2025 Nielsen VM et al. *JAMA Network Open*

eFigure 1. Directed Acyclic Graph.

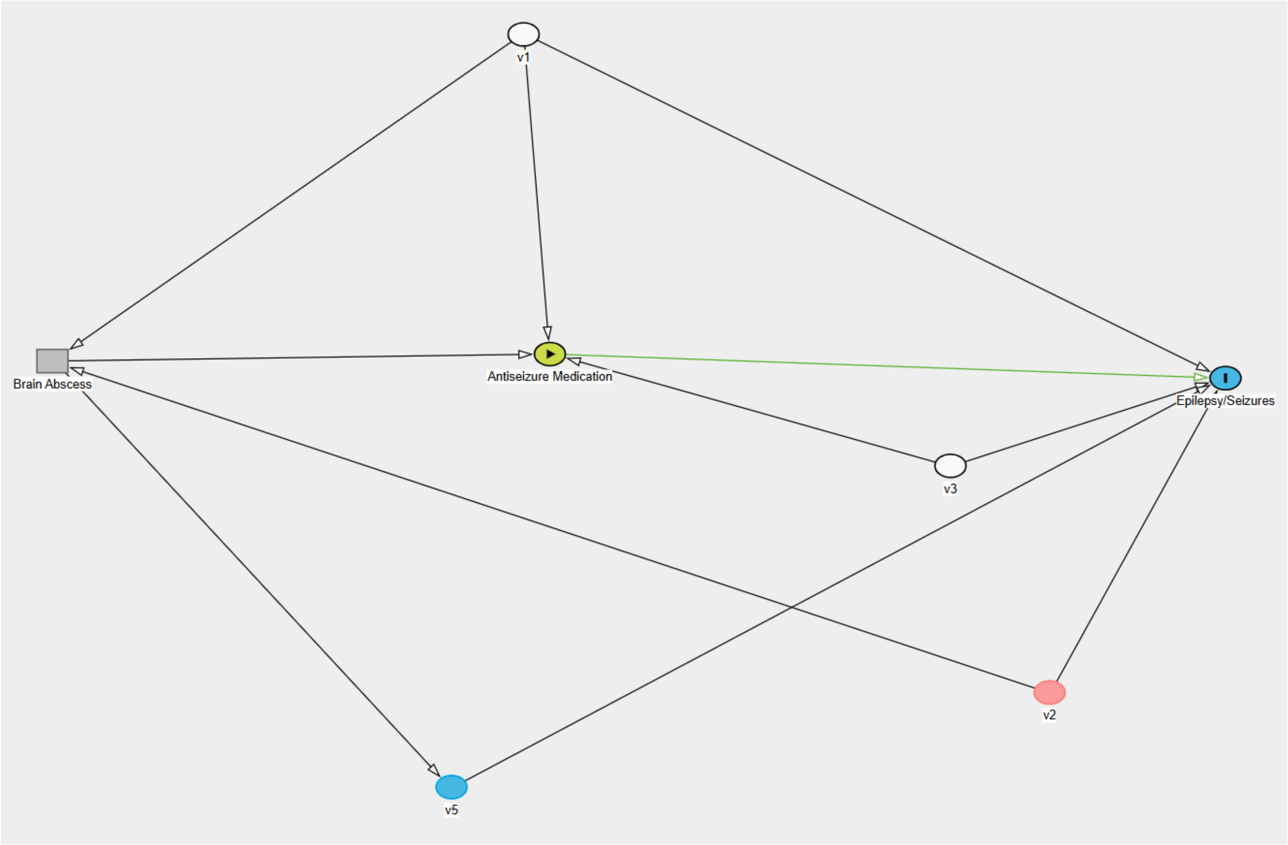

v1 – Variables causal or associated with brain abscess, antiepileptic drug initiation, and epilepsy: traumatic brain injury, congenital heart disease, stroke, alcohol misuse, brain cancer, prior neurosurgery, Charlson Comorbidity Index, sex.
v3 – Variables causal or associated with antiepileptic drug initiation and epilepsy: early seizures, sepsis, critical illness, neurosurgical management of abscess (i.e., craniotomy, aspiration), age.

eMethods. Detailed Statistical Analysis Plan.

We applied inclusion and exclusion criteria outlined under the Population section of this manuscript, resulting in 572 patients eligible for the analysis. This appendix describes analysis of the main study outcome only (occurrence of epilepsy after 14 days with 45-day grace period). However, full model diagnostics were repeated for all sensitivity analyses.

Preparation of the Data
We cloned all 572 patients and assigned one clone to the control arm and one clone to the treatment arm. In the control arm, the clone was censored if they initiated an AED within the 45-day grace period. In the treatment arm, the clone was censored if they completed 45-day grace period without starting an AED. Thus, in the control arm, a patient's total follow up time was coded as the date they initiated the drug (censored) or, if they did not start the AED within 45 days, had a seizure or were lost to follow up, whichever came first. In the treatment arm, a patient's follow up time was set to 45 days if they did not start an AED within 45 days (censored), or, if they did initiate AED within 45 days, had a seizure or were lost to follow up, whichever came first.

This approach results in all patients contributing data to both arms if they did not survive long enough to begin treatment (either due to loss to follow up or having the outcome). Once the 45-day grace period is over and if they remain uncensored in their respective study arm, they contribute follow up to the subsequent outcome model. Finally, we split the data such that we created an observation for each day that a time-varying event occurred, up until that patient's follow up time ended.

Generation of Inverse Probability Weights
We ran a Cox proportional hazards model for the control group and a logistic regression for the treatment group (as censoring in the treatment group could only occur at one time point) of the 45-day grace period using censoring as the event. These models generated the inverse probability weights for the subsequent analysis models. We used Efron's approximation to handle ties. We included covariables listed in the main manuscript. All variables were coded as binary, except for CCI and age, which were treated as continuous. Time-varying covariables were carried forward through time once they occurred.

We evaluated each model fully to ensure adherence to model assumptions, including outliers (DFBETA), violation of the proportional hazard assumption (Cox model only), log-linear relationship between the outcome and continuous covariables, and multicollinearity. The model for the treatment group suggested no violations of model assumptions. The Cox model for the control group indicated violations of proportional hazards for critical illness, stroke, and craniotomy of the abscess. We thus stratified on these covariables. A stratified model further assumes that the hazards of coefficients are the same between strata – we tested this with all possible interactions between each covariable and each stratum and assessed model fit with Akaike Information Criterion (AIC). AIC was reduced with inclusion of an interaction term between the critical illness stratum and aspiration of abscess (stratified model AIC = 804.3; stratified model with interaction AIC = 757.6). We thus retained the stratified model with the interaction term. All other model assumptions were met.

We then estimated inverse probability weights from the treatment and control Cox models using the following methods. We generated linear predictions for each observation by multiplying each patient's covariable values by the beta estimates from the model. We then estimated the baseline hazard for each model. Note that in the control Cox model, the baseline hazard varies by stratum. Thus, we generated a unique baseline hazard for each stratum from the control Cox model and joined based on the patient's covariable values of critical illness, history of stroke, and history of traumatic brain injury. We then multiplied the negative exponentiated baseline hazard with the exponentiated linear prediction. We then took the inverse of this value, resulting in the final inverse probability weight.

Assessment of Inverse Probability Weights

© 2025 Nielsen VM et al. *JAMA Network Open*

We assessed model weights for extreme values and sufficient overlap between the treatment group and the control group. Insufficient overlap and extreme weights may reflect positivity violations. The mean weight in the treated and control arms were similar (treated: 1.81, standard deviation [SD] 2.24; control: 1.17, SD 0.21). Both demonstrated good overlap, with both groups having a right skewed tail (**eFigure 2**). Although the treated group had a larger maximum weight than the control group, the majority of weights in both groups were less than 5. Assessment of standardized mean differences (SMD) before and after weighting suggested good balance of confounders (shown in main manuscript).

© 2025 Nielsen VM et al. *JAMA Network Open*

eFigure 2. Inverse Probability Weights in Treatment and Control Arms.

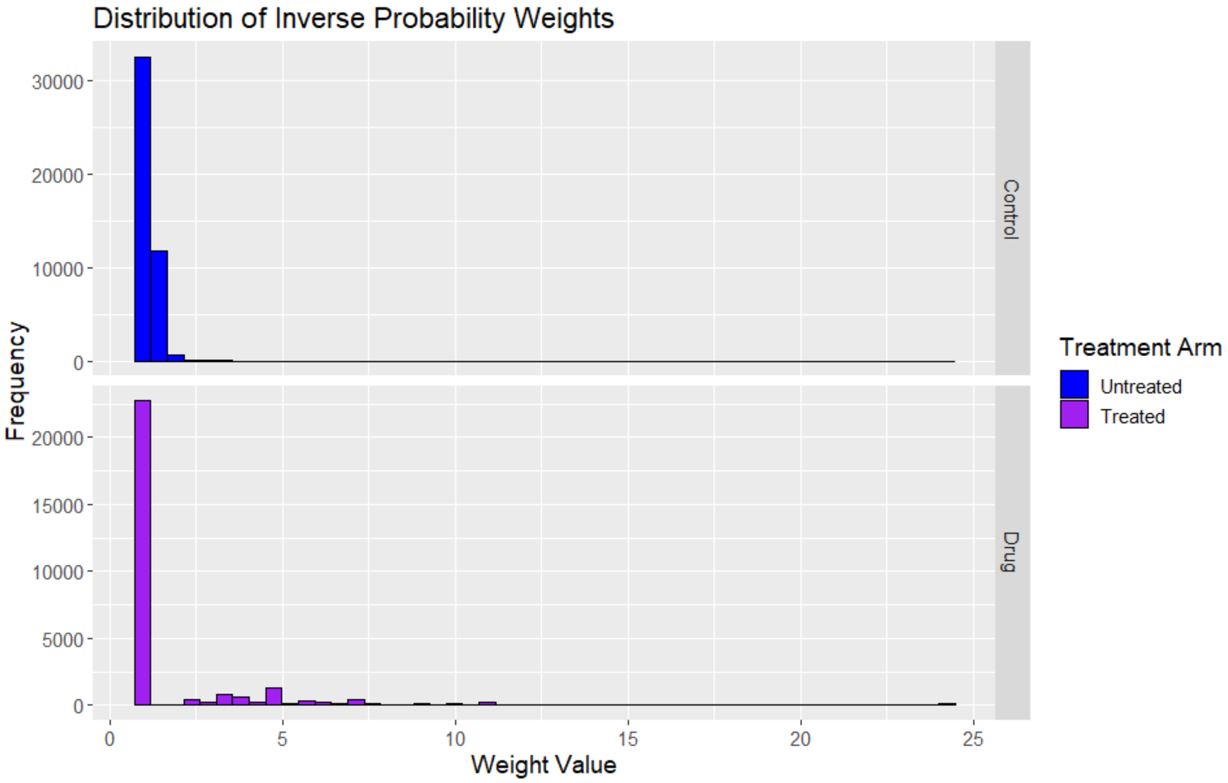

© 2025 Nielsen VM et al. *JAMA Network Open*
