# Supplementary material for: Initiation of Antiseizure Medications in Patients With Brain Abscess
Source: JAMA Netw Open. 2025 Aug 1;8(8):e2524557. doi: 10.1001/jamanetworkopen.2025.24557 (PMC12317356; doi:10.1001/jamanetworkopen.2025.24557)
Supplement: Supplement 2. — Data Sharing Statement [file jamanetwopen-e2524557-s002.pdf]

Data Sharing Statement

Nielsen. Initiation of Antiseizure Medications in Patients With Brain Abscess. *JAMA Netw Open*. Published August 01, 2025. doi:10.1001/jamanetworkopen.2025.24557

Data
Data available: No

Additional Information
Explanation for why data not available: The authors are not owners of the PharMetrics data and are not licensed to share it. Qualified researchers may obtain access to the PharMetrics data through IQVIA (https://www.iqvia.com).
